# Evaluation of the immune system status and hematological dyscrasias, among amphetamine and cannabis abusers at Eradah Hospital in Qassim, Saudi Arabia

**DOI:** 10.1038/s41598-024-61182-4

**Published:** 2024-05-08

**Authors:** Amal Hussain Mohammed, Atheer Saleh Aljarallah, Mohsina Huq, Amal M. H. Mackawy, Basmah F. Alharbi, Khulud Salem Almutairi, Abdulmohsen M. Alruwetei, Ahmad Abdulaziz A. Almatroudi, Hajed Obaid Alharbi, Said Abdel Mohsen A. Aljohery, Afshan Zeeshan Wasti

**Affiliations:** 1 Department of Medical Laboratories, College of Applied Medical Sciences, Qassim University, 51452 Buraydah, Saudi Arabia; 2 Department of Basic Health Sciences, College of Applied Medical Sciences, Qassim University, 51452 Buraydah, Saudi Arabia; 3 Department of Biochemistry, Jinnah University for Women, Karachi, 74600 Pakistan; 4 Medical Biochemistry and Molecular Biology, Faculty of Medicine, Zagazig University, Zagazig, Egypt; 5 Director Laboratory Department, MOH-KSA, Erada Mental Health Hospital, Buraydah, Qassim Saudi Arabia; 6 Faculty of Medicine, Zagazig University, Zagazig, Egypt

**Keywords:** Amphetamine, Cannabis, Immune system, Fungal infection, Toxicology, Immunology, Microbiology

## Abstract

This cross-sectional study aims to evaluate the immune system status and hematological disturbances among individuals who abuse amphetamines and cannabis. Substance abuse, particularly of amphetamines and cannabis, has been associated with various adverse effects on the body, including potential impacts on the immune system and hematological parameters. However, limited research has been conducted to comprehensively assess these effects in a cross-sectional design. Additionally, fungal infections are on the rise internationally, and immune-compromised people are particularly susceptible. The study will recruit a sample of amphetamine and cannabis abusers (n = 50) at the Eradah Hospital in the Qassim Region of Buraydah and assess their sociodemographic and biochemical variables, including blood indices and differential WBC indices, liver, and kidney profiles. Additionally, 50 sputum samples in total were cultured for testing for fungus infections. To obtain the descriptive statistics, the data was imported into Microsoft Excel and subjected to statistical analysis using SPSS 22.0. Amphetamine and cannabis abuser's sociodemographic variables analysis observed that the majority (52%) were aged 18–30, with 56% in secondary school. Unemployment was a significant issue, and most had no other health issues. The majority (50%) had 5–10 years of abuse, while 32% had less than 5 years, and only 18% had been drug abusers for more than 10 years. There were significant changes (*p* < 0.001) in all different leukocyte blood cells, including neutrophils, lymphocytes, monocytes, eosinophils, and basophils. Furthermore, a microscopic examination of blood films from individuals who misuse the combination of the medications "amphetamine and cannabis" reveals hazardous alterations in Neutrophils. Out of 50, 35 sputum samples showed positive growth on Sabouraud dextrose agar (SDA) with chloramphenicol antibiotic, indicating a unicellular fungal growth. The present study explores the immune system and hematological disturbances linked to amphetamine and cannabis abuse, providing insights into health risks and targeted interventions. The findings complement previous research on drug users' hematological abnormalities, particularly in white blood cells. Routine hematological tests help identify alterations in homeostatic conditions, improving patient knowledge and preventing major issues. Further research is needed on multi-drug abuse prevention, early detection, and intervention. The cross-sectional design allows for a snapshot of the immune system and hematological status among abusers, laying the groundwork for future longitudinal studies. Key Words: Drug Effect, Immunity, Epidemiology, Oxidative Stress, Inflammation.

## Introduction

Amphetamine and cannabis abuse are an alarming issue worldwide, with significant implications for public health. Addiction negatively impacts individuals and society, leading to crimes. Saudi Arabia's General Directorate for Drug Control combats addiction by swiftly implementing measures, controlling smugglers, and raising awareness among citizens, particularly young people, about the harms of drugs^1^.

Important medications with stimulant and anxiolytic properties frequently are misused or overused, which has been linked to serious side effects, and higher mortality and morbidity rates around the world, and severe side effects. Cannabis and amphetamine are two narcotics that are frequently combined, and addiction to both drugs has risen globally, especially in Saudi Arabia^2^. In addition, a study in Jazan, Saudi Arabia found that the number of fatalities related to amphetamine addiction and other narcotics substances increased from 18 to 80% between 2018 and 2020^3^, but in the Qassim region, cannabis addiction was the most prevalent drug usage, followed by amphetamine^2^.

These substances have psychoactive properties and are commonly used for recreational purposes. However, their abuse can lead to various adverse effects on the body, including disturbances in blood indices, liver function, and kidney profile. Understanding the impact of amphetamine and cannabis abuse on these physiological parameters is crucial for assessing the overall health risks associated with substance abuse. Amphetamine and cannabis abuse have been linked to alterations in blood indices. Studies have reported changes in red blood cell count, white blood cell count, and platelet count among individuals who abuse these substances^4,5^. These alterations can have implications for overall blood health and may increase the risk of hematological disorders.

The liver plays a vital role in metabolizing drugs and toxins, including amphetamines and cannabis. Prolonged abuse of these substances can lead to liver damage and dysfunction. Elevated liver enzymes, such as alanine aminotransferase (ALT) and aspartate aminotransferase (AST), have been observed in individuals with amphetamine and cannabis abuse^2,6^. These findings suggest potential hepatotoxic effects associated with substance abuse.

Amphetamine and cannabis abuse have been associated with renal dysfunction and impaired kidney function. Studies have reported increased levels of serum creatinine and blood urea nitrogen (BUN) in individuals who abuse these substances^7,8^. These findings indicate potential nephrotoxic effects associated with substance abuse. Chronic amphetamine abuse disrupts the release of cytokines in microglial cells, causing inflammation and suppressing immune responses. This can lead to liver function changes. Studies have shown a significant reduction in serum immunoglobulin levels among amphetamine abusers, potentially causing immunodeficiency with recurrent infections^9,10^.

In addition, another study found that there is leukopenia including granulocyte and monocyte, induced by prolonged psychostimulant treatment. Recently studies indicate amphetamine stands for impact on both innate and adaptive immune systems and amphetamine abusers will stay immunocompromised even after treatment^11^.

Cannabis abuse can cause health issues like respiratory complications, immune system dysfunction, and increased infection susceptibility. Tetrahydrocannabinol, a widely studied cannabinoid, is responsible for most effects through brain-specific receptors. Marijuana, derived from *Cannabis sativa* leaves, contains significant quantities of THC, which is responsible for mental state effects among users^12^. Furthermore, cannabis has neurological impacts and alters cytokine levels, triggering immune modulation. It reduces pro-inflammatory cytokines and raises anti-inflammatory ones. Maternal exposure to cannabis during pregnancy can cause dysregulation of the fetus's innate and adaptive immune systems, weakening its defense against infections and cancers^13,14^.

Oxidative stress is a biological imbalance resulting from the excess production and accumulation of oxygen-reactive species (ROS) in cells and tissues, which can cause cell and tissue damage due to environmental stressors and xenobiotics. Caused by drug-induced damage to vital organs like the liver, kidney, cardiovascular, and nervous systems, results from mitochondrial dysfunction and dopamine oxidation. This leads to deterioration of cell components and a pro-apoptotic response. Abusers of amphetamine and cannabis increase serum oxidative stress biomarkers, disrupting immune system functions, and leading to infections and inflammation^15–17^.

Primarily, most labs test for substances that are often abused. Depending on the drug and the mode of delivery, blood tests can be used to identify drug use^18^. Furthermore, there is a growing focus on the hematological abnormalities that substance addicts experience, which may have serious medical repercussions. There are direct hematological adverse effects of acute and chronic drug abuse, including toxic effects on bone marrow or other blood-forming organs, leading to lower-than-normal or malfunctioning blood cells. Indirect effects may lead to alterations in metabolic and/or physiological functions, which determine liver and kidney diseases, and nutritional deficiencies, that affect the function of various blood cells^19,20^.

Factors contributing to the increase in fungal infections include aging immunity, pulmonary diseases, and immunosuppressive regimes. The World Health Organization (WHO) has published a list of nineteen fungal priority pathogens, highlighting high-risk diseases. However, less is known about newer risk factors, and misdiagnosis and poor understanding by healthcare providers make it difficult to determine the global burden, leading to potentially more infected people than currently believed^21^.

This cross-sectional study aims to evaluate amphetamine and cannabis drug-related toxicity in immunocompromised drug users by measuring different biochemical parameters such as the blood indices, differential WBCs indices, and liver and kidney profiles. Also, susceptibility and prevalence of fungal infections, among these drug abusers particularly in the Qassim region, KSA.

## Methodology

### Study design and participants

A cross-sectional study was carried out at the Eradah Hospital in Qassim, Saudi Arabia. Volunteer sampling techniques were employed to recruit Saudi males and females aged 18 years or older who abuse cannabis and amphetamines. This study aims to gather samples from 50 participants, according to the DSM-V criteria for drug abuse, describing the continued use of cannabis despite impairment in psychological, physical, or social functioning. Inclusion criteria encompass Saudi female and male participants aged 18 or above who consent to participate. Exclusion criteria involve non-Saudi individuals, participants under 18 years old, and those who decline participation.

### Informed consent

Each participant's permission was also sought after being informed of the full scope of the study's goals and the procedures used to collect the samples. However, the participant's informed consent was obtained with the doctor's supervision, and the information gathered was kept confidential and used solely for study.

### Sample size calculation

The sample size estimation for a cross-sectional study with one group is calculated using the formula: n = Z^2 (1 − a/2) × P (1 − P)/d^2. Here, n represents the sample size, Z^2 (1 − a/2) is the confidence interval (1.65 for 95% confidence), P is the estimated proportion (0.041 for the proportion of individuals abusing cannabis and amphetamines), and d is the desired precision (0.05). Plugging in the values, we get n = 1.65^2 × 0.041 (1–0.041)/0.05^2, which simplifies to n = 2.7225 × 0.0393/0.0025. Further simplification yields n = 0.107/0.0025, resulting in n = 42.8. Therefore, the estimated number of research participants is approximately 43 individuals who abuse cannabis and amphetamines. Rounded up, this suggests a sample size of fifty participants.

### Sample collection and biochemical analysis

Three ml of whole blood samples were taken in Ethylenediaminetetraacetic acid (EDTA) tubes, which were drawn by the nurses in the hospital to measure the Complete Blood Count (CBC) with blood differential by “Sysmex xn series” for these parameters (WBC, RBC, HB, HCT, MCV, MCH, MCHC, PLT, NE, LYM, MON, EO, BASO), and then also blood film observed under a microscope (Light microscope (OLYMPUS), to look at leukocyte cell structure. The prepared blood films were stained with 10% Giemsa stain for 10 min after completion of fixation by methanol. Additionally, the “Dimension & Cobas” (Roche, Switzerland) machine used blood samples in a heparin tube for testing ALT, AST, ALP, Creatinine, and BUN to check the kidney and liver functions among participants. Before being processed in automated analyzers, the blood samples underwent centrifugation (1500 rpm for 10 min) to separate their constituents, yielding clear plasma for precise and reliable results. The conducted tests were executed in strict compliance with established quality control standards, encompassing the calibration and maintenance procedures of utilized equipment to uphold precision and reliability in the obtained results. Furthermore, adherence to laboratory safety protocols was maintained, along with the sustenance of a hygienic and systematically arranged operational setting. After the observation and collection of results, appropriate disposal measures were undertaken, with all samples handled with precision. Contaminated cultures and pipettes were specifically disposed of in impermeable red bag containers bearing biohazard symbols to ensure proper containment.

To quantify immunological function in amphetamine and cannabis users and screen the sample for fungal infections, the study calls for the collection of sputum samples. Participants cough up sputum samples from deep within the chest into a sterile plastic collection bottle, which are then cultivated on Sabouraud dextrose agar with Chloramphenicol, at 25 °C for one week of incubation in a hospital microbiology lab. After incubation, fungi were identified through macroscopic examination by observing the colony morphology, color, texture, and growth pattern which provide initial clues about the fungi present. Further, the microscopic examination was conducted using a stain (Crystal violet), to analyze the fungal structures. Before commencing a culture, the suitability of a sputum sample for fungal analysis was substantiated, ensuring it exhibits purulent characteristics indicative of lower respiratory tract origin and contains a sufficient quantity for culturing purposes, while also being free from saliva contamination.

### Statistical analysis

Microsoft Excel 2023 was used to input, tabulate, and analyze the gathered data. The quantity and percentage of categorical variables were determined to apply the descriptive analysis. To handle and analyze more data, the SPSS 22.0 application was used. The measured data distribution was represented by mean ± SD. The investigation of the link among the different factors was conducted by basic correlation analysis. The ANOVA test was employed to examine the average difference between the two groups under investigation. When the P value is less than 0.05, the correlation and difference between variables are deemed statistically significant.

### Ethical considerations

Study approval was obtained from the Regional Research Ethics Committee, Registered at the National Committee of Bio & Med. Ethics [NCBE] Registration No. H-04-Q-001. Participants were allowed to provide informed consent before sample collection and were briefed on the study's objectives. Each participant completed informed consent forms, explicitly acknowledging their ability to withdraw from participation at any given time. Strict confidentiality measures were implemented to protect the dataset for research purposes only, ensuring participant anonymity by omitting sensitive identifiers such as names and addresses. All methods were performed according to the relevant guidelines and regulations, particularly with the Declaration of Helsinki, which states the ethical standards for medical research involving human beings.

### Potential biases and limitations

In this cross-sectional study with a volunteer sampling technique, both sampling bias and confounding variables presented challenges. Participants self-selected, potentially distorting the population's view by overrepresenting or excluding certain groups. Additionally, confounding variables made it difficult to accurately attribute observed associations, limiting the study's ability to draw meaningful conclusions about cause and effect due to the lack of random assignment or control.

## Results

The sociodemographic variables of the 50 amphetamine and cannabis abusers' participants were included in this study (Table 1). For age, it is noticed that the majority, about half (52%), were 18–30 years of age, fewer (36%) were 31–40 years of age, and only 12% were above the age of 41 years. Most of the abusers (56%) are in secondary school-level education; a few (14%) were in preparatory school, and a few (4%) were in elementary school, whereas 26 percent were college-educated. In addition, only 24% of abusers answered that they have other health issues, and 76% answered no. The data collected from the abusers about the number of years of addiction shows the following: 32% are less than 5 years, 50% are from 5 to 10 years, and 18% are more than 10 years. Whereas 92% of the abusers are unemployed and 8% are employed.

**Table 1:** Distribution of Cannabis and Amphetamine abusers based on their demographic characteristics.

Demographic variables	Cannabis and amphetamine	
	Count	%
Age		
18–30	26	52
31–40	18	36
41 and above	6	12
Educational level		
Elementary school	2	4
Preparatory school	7	14
Secondary school	28	56
College grade	13	26
Other health issues		
No	38	76
Yes	12	24
Duration of abuse		
Less than 5 years	16	32
From 5 to 10 years	25	50
More than 10 years	9	18
Employee		
No	46	92
Yes	4	8

Table 2 presents a comprehensive hematological analysis of amphetamine and cannabis abusers (n = 50). For this study, the analysis of various blood cell counts and indices means, standard deviations, t-tests, and *p* values for hypothesis testing between the “normal” and “non-normal” groups. For instance, “Norm” which means normal range or values, and “Non-Norm” which means abnormal values to compare the biochemical parameters. The findings provide valuable insights into the distribution and significance of blood parameters between normal and non-normal categories, contributing to our understanding of the physiological variations in these parameters.

**Table 2:** Complete Blood Count (CBC) analyzed results for Cannabis and Amphetamine abusers.

Blood indices	N	Mean	SD	Categories	N	Mean	SD	Student’s t-test	*p*
RBC	50	5.07	0.44	Norm	50	5.0684	0.44422	–	–
4–6.5				Non-norm	0	.	.		
HB	50	14.63	1.28	Norm	45	14.8844	1.0514	5.236	0.001
13–17				Non-norm	5	12.34	0.77006		
HCT	50	44.24	3.53	Norm	46	44.6109	2.74066	1.131	0.339
37–52				Non-norm	4	40.025	8.07026		
MCV	50	87.18	4.68	Norm	47	88.0426	3.15407	7.637	0.001
78–95				Non-norm	3	73.6	3.63868		
MCH	50	29.03	1.93	Norm	47	29.4128	1.19357	9.072	0.001
26–32				Non-norm	3	23.0333	0.83865		
MCHC	50	33.34	1.05	Norm	44	33.5909	0.82682	5.983	0.001
32–36				Non-norm	6	31.5	0.55857		
PLT	50	252.24	51.33	Norm	49	254.49	49.3048	2.259	0.028
150–450				Non-norm	1	142	.		
WBC	50	7.55	1.82	Norm	47	7.3049	1.57759	− 16.213	0.001
4–10				Non-norm	3	11.3967	0.17954		
NE	50	2.93	1.33	Norm	39	3.2741	1.2787	7.182	0.001
2–7.5				Non-norm	11	1.6709	0.29484		
LYM	50	2.81	0.98	Norm	37	3.2241	0.77231	11.617	0.001
2–7.5				Non-norm	13	1.6185	0.19693		
MON	50	0.73	0.39	Norm	42	0.5988	0.1766	− 4.569	0.002
0.2–1				Non-norm	8	1.4063	0.49387		
EO	50	0.25	0.17	Norm	14	0.4814	0.11786	9.388	0.001
0.3–1				Non-norm	36	0.1636	0.07442		
BASO	50	0.04	0.02	Norm	2	0.1	0	4.689	0.001
0.1–0.3				Non-norm	48	0.0342	0.01966		

*Significance, if present, is in favor of the category with the highest arithmetic mean, **Correlation is significant at *p* < 0.001.

Our study also shows significant changes in different white blood cell types, including neutrophils (NE; *p* value = 0.001), lymphocytes (LYM; *p* value = 0.001), monocytes (MON; p-value = 0.002), eosinophils (EO; *p* value = 0.001), and basophils (BASO; *p* value = 0.001), suggesting immune system disorder. Whereas, there are no significant changes for RBC, HCT, RBC indices, or PLT (Table 2).

**Figure 1:**
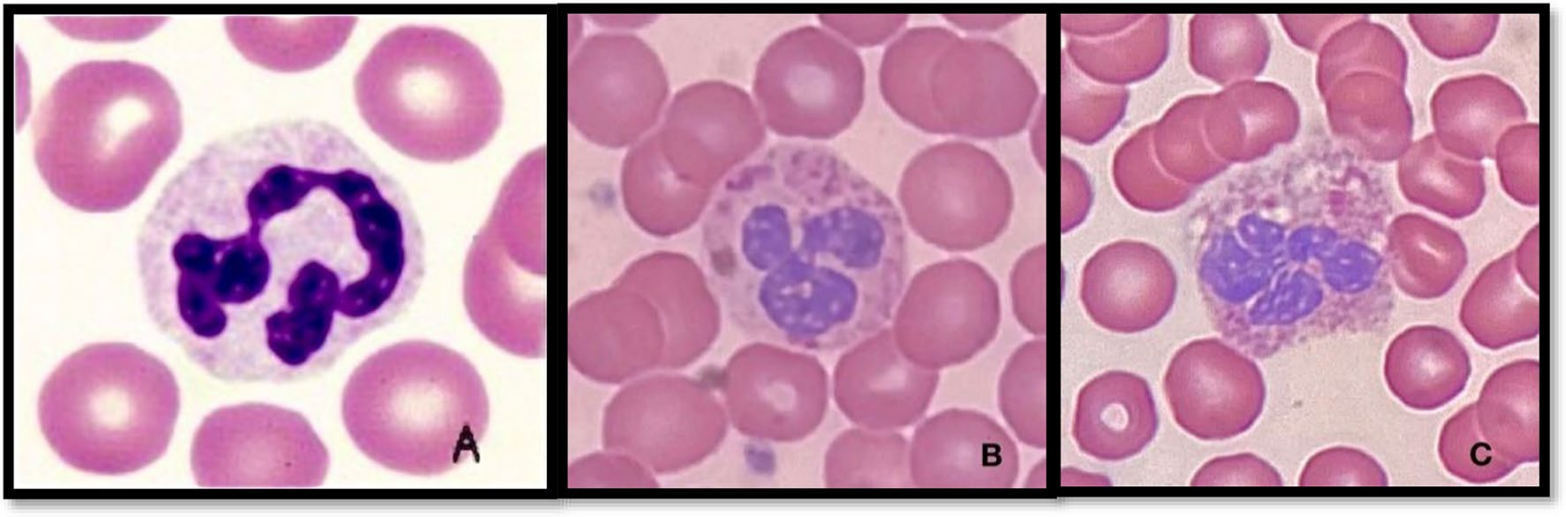
The microscopic view of blood film for patients who abuse the mixing drugs “Amphetamine and Cannabis”. (**A**) Normal neutrophils. (**B**–**C**) Neutrophils with toxic change.

The study's most startling discovery is that amphetamine and cannabis abusers' peripheral blood smears frequently exhibit neutrophil cytotoxicity. We can interpret white blood cell properties (size, shape, and color) by looking into peripheral blood films. Panel A displays the typical features, while Panels B and C display harmful alterations (Fig. 1). During differential counting, the neutrophil showed cytotoxic impact, including nuclear crumbling, vacuolization, and defragmentation, which may suggest the impact of drug abuse involving some cytotoxic metabolites such as free radicals, ROS- reactive oxygen species, and cytokines, and specifically the vacuolization in neutrophils is strongly representative of infection.

The study analyzed the impact of cannabis and amphetamine addiction on the liver and renal profiles (Table 3). Results showed a significant positive link between years of abuse and the liver profile (ALT-GPT), with a correlation coefficient of r = 0.453. The study also found a significant effect of years of abuse on ALT-GPT, accounting for 21% of the variation. The data also showed a positive relationship between years of abuse and AST-GOT, with a correlation coefficient of r = 0.641, suggesting that abuse over years accounts for 41% of the variation in AST-GOT. However, there was no statistically significant effect of years of misuse on alkaline phosphatase, with a *p* > 0.196.

**Table 3:** Simple linear regression analysis test of the effect of the number of years of Amphetamines and Cannabis abuse on different parameters of LFTs, and KFTs profile.

No. of years of drug abuse	B (beta)	(F)	*p* value	Correlation (R)	Coefficient of determination (R2)
ALT-GPT	1.848	12.387	0.001	0.453	0.205
AST-GOT	1.574	33.448	0.001	0.641	0.411
ALP	0.488	1.719	0.196	0.186	0.035
Creatinine	0.711	6.95	0.011	0.356	0.121
BUN	0.081	6.637	0.013	0.349	0.121

*ALT-GPT* alanine aminotransferase-glutamic pyruvic transaminase, *AST-GOT* aspartate aminotransferase-glutamic oxaloacetic transaminase, *ALP* alkaline phosphatase, *BUN* blood urea nitrogen.

*Correlation is significant at *p* < 0.001.

Furthermore, there is a statistically significant and positive correlation between creatinine and BUN and several years of abuse. At a significance threshold of α ≤ 0.05, the correlation coefficient of 0.356 indicates that years of abuse had a substantial impact on creatinine. The *p* > 0.011, and the coefficient of determination (r^2^) is 0.121. This implies that 12% of the fluctuation in creatinine over the years can be attributed to drug misuse. Additionally, the study demonstrates a substantial influence on both variables and a positive correlation between the number of years of abuse and BUN.

To check differences in patients who take the two types of drugs and demographic variables, an ANOVA test has been used, where the null hypothesis refers to the fact that there are no significant differences in patients who take the two types of drugs and demographic variables represented in Table 4. There were established significant differences in MONO related to the age variable and blood variables, where the p-value of the test was 0.035 less than 0.05. Whereas, other significant differences were established in ALT-GPT and AST-GOT related to age variables and liver variables, where the p-values of these tests were 0.000 and 0.001, respectively. Therefore, the results conclude that the age variable affects the emergence and rate of the impact of drug abuse. However, this correlation does not imply causation, meaning it only refers to the variables solely based on an observed association or correlation between them. Additionally, there are no significant differences in other demographic variables correlated with blood variables, liver variables, or kidney variables.

**Table 4:** Correlation analysis of Age variables with CBC, LFTs, and KFTs in Cannabis and Amphetamine abusers.

Variables	Age (years)	N	Mean	Std. Dev	F	Sig*
WBC	18–30	26	7.606	2.032	0.358	0.701
	31–40	18	7.313	1.778		
	41 and above	6	8.022	0.681		
RBC	18–30	26	5.072	0.434	0.303	0.740
	31–40	18	5.023	0.487		
	41 and above	6	5.188	0.404		
HGB	18–30	26	14.788	1.079	0.692	0.505
	31–40	18	14.344	1.585		
	41 and above	6	14.800	1.101		
HCT	18–30	26	44.535	3.174	0.519	0.599
	31–40	18	43.583	4.398		
	41 and above	6	44.967	1.838		
MCV	18–30	26	87.142	4.656	0.005	0.995
	31–40	18	87.261	3.123		
	41 and above	6	87.067	8.535		
MCH	18–30	26	29.162	1.845	0.181	0.835
	31–40	18	28.967	1.689		
	41 and above	6	28.650	3.055		
MCHC	18–30	26	33.485	0.810	0.585	0.561
	31–40	18	33.233	1.213		
	41 and above	6	33.033	1.503		
PLT	18–30	26	245.038	40.087	0.768	0.470
	31–40	18	255.833	53.481		
	41 and above	6	272.667	85.092		
NE#	18–30	25	3.285	1.673	1.895	0.162
	31–40	18	2.602	0.733		
	41 and above	6	2.452	0.640		
LYM#	18–30	26	2.788	0.978	0.215	0.807
	31–40	18	2.752	1.031		
	41 and above	6	3.052	0.933		
MONO#	18–30	26	0.605	0.194	3.592	0.035*
	31–40	18	0.813	0.515		
	41 and above	6	1.003	0.436		
EO#	18–30	26	0.258	0.167	0.837	0.439
	31–40	18	0.222	0.155		
	41 and above	6	0.323	0.218		
BASO#	18–30	26	0.038	0.022	0.361	0.699
	31–40	18	0.033	0.025		
	41 and above	6	0.042	0.026		
ALT-GPT	18–30	26	2.00	0.000	20.680	0.000*
	31–40	18	2.00	0.000		
	41 and above	6	2.50	0.548		
AST-GOT	18–30	26	1.69	0.471	8.778	0.001*
	31–40	18	2.00	0.485		
	41 and above	6	2.67	0.816		
ALP	18–30	26	2.00	0.000		
	31–40	18	2.00	0.000		
	41 and above	6	2.00	0.000		
CREATININE	18–30	26	1.96	0.196	0.451	0.640
	31–40	18	2.00	0.000		
	41 and above	6	2.00	0.000		
BUN	18–30	26	2.00	0.000	1.880	0.164
	31–40	18	2.06	0.236		
	41 and above	6	2.17	0.408		

*Significance, if present, is in favor of the category with the highest arithmetic mean, *Correlation is significant at *p* < 0.001.

**Figure 2:**
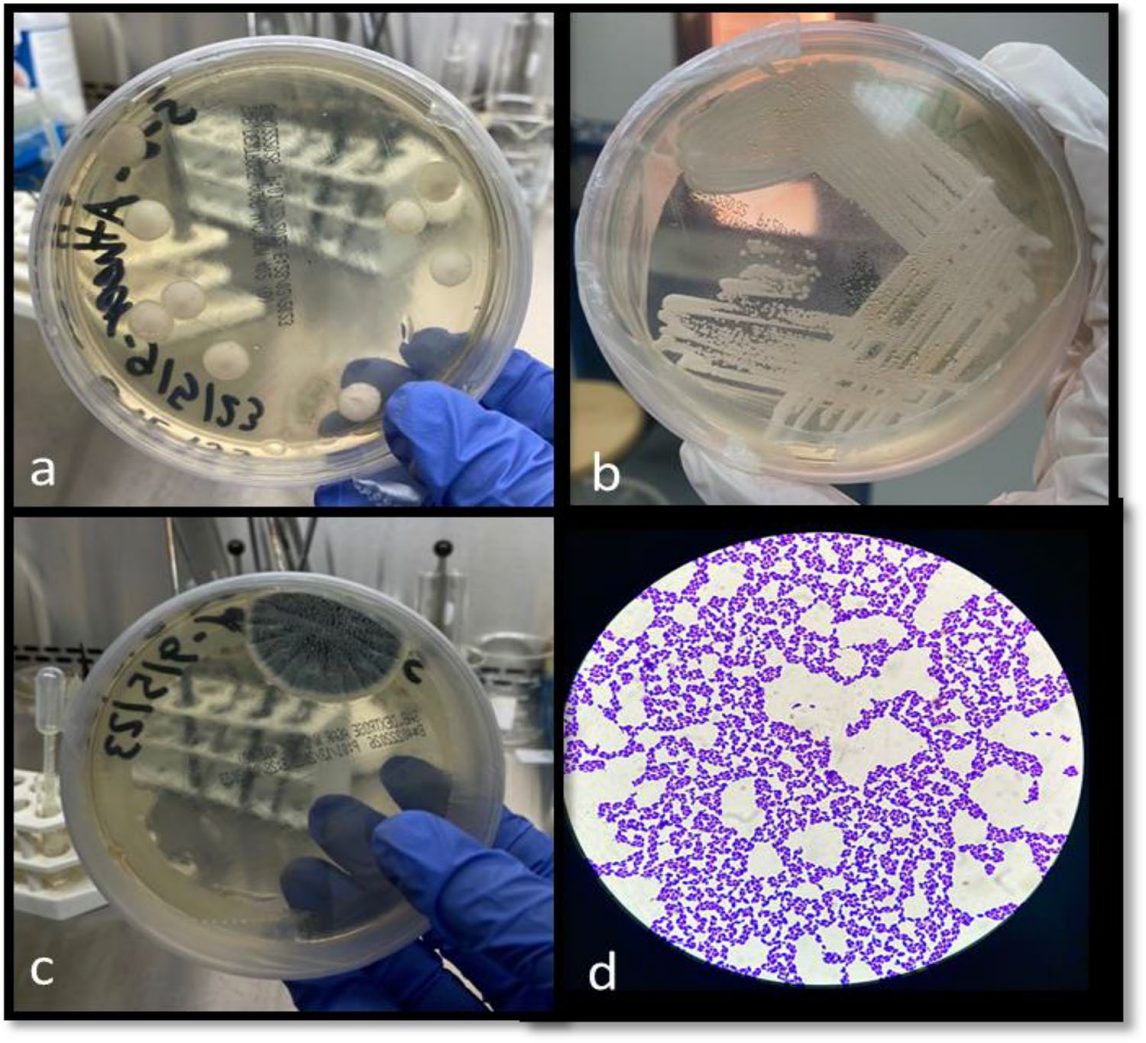
(**a**,**b**) Positive growth of fungi on Sabouraud dextrose agar (SDA). (**c**) A growth of mold. (**d**) Microscopic view of yeast with crystal violet stain shows small oval-shaped cells with budding.

A total of 50 sputum samples were cultured on “Sabouraud dextrose agar (SDA) with chloramphenicol” antibiotic to prevent the growth of a variety of Gram-positive and Gram-negative bacteria (Fig. 2a–c). After one week of incubation at 25 °C, the majority of the samples (35 cultures) recorded positive growth, while 15 cultures recorded negative growth (Table 5). Moreover, some of the positive cultures are then stained with “Crystal Violet” and examined under the microscope. Figure 2d shows a single or cluster of oval-shaped cells with budding characteristics, indicating a unicellular fungus. However, further studies should be performed to identify the specific type.

**Table 5:** Fungal culture test of Cannabis and amphetamine abuser’s sputum samples on Sabouraud dextrose agar (SDA).

		COUNT	%
SDA	Positive	35	70
	Negative	15	30

Sample number	Fungal growth	Sample number	Fungal growth
1	+, yeast	26	+, yeast
2	–	27	–
3	+, yeast	28	+, yeast
4	+, yeast	29	+, yeast
5	–	30	+, yeast
6	+, yeast	31	+, yeast
7	+, mold	32	+, yeast
8	–	33	–
9	+, yeast	34	+, yeast
10	+, yeast	35	+, yeast
11	+, yeast	36	–
12	–	37	+, yeast
13	–	38	+, yeast
14	–	39	+, yeast
15	+, yeast	40	+, yeast
16	–	41	+, yeast
17	–	42	+, yeast
18	+, yeast	43	+, yeast
19	+, yeast	44	+, yeast
20	+, yeast	45	+, yeast
21	+, yeast	46	+, yeast
22	–	47	+, yeast
23	–	48	+, yeast
24	+, yeast	49	–
25	–	50	+, yeast

## Discussion

Amphetamine and cannabis are frequently abused substances, and experimental evidence shows that cannabis use is associated with negative risks for non-medical amphetamine users^22^. The purpose of this cross-sectional study is to assess the effects of cannabis and amphetamines on various sociodemographic and biochemical characteristics in individuals who abuse, specifically in the Qassim region of Saudi Arabia, and may not be generalizable to other populations due to cultural, environmental, and genetic differences that can influence substance abuse patterns and health outcomes.

The sociodemographic variables of the 50 amphetamine plus cannabis abusers' participants in this study (Table 1). For age, it is noticed that the majority, about half (52%), were 18–30 years of age, fewer (36%) were 31–40 years of age, and only 12 percent were above the age of 41 years. Most of the abusers (56%) are in secondary school-level education; a few (14%) were in preparatory school, and a few (4%) were in elementary school, whereas 26 percent were college-educated. It may suggest that adolescents often experiment with drugs due to rapid physical and intellectual development, which can be influenced by life events and social group affiliations, leading to an experimental attitude and increased drug use risk^23–25^.

A significant difference was observed in the employee ratio, where a considerable number of abusers are unemployed. Our findings are supported by a previous study^26^ that revealed that people who were jobless and had the lowest degree of education were more likely to be heavy drinkers, smokers, and physically inactive. Other variables of the present study were whether the participants had other health issues, and the majority of the answers were no. Lastly, the number of years of abuse that constituted 50 percent were from 5 to 10 years, slightly less (32%) was less than 5 years, and just 18% were drug abusers for more than 10 years.

Chronic abuse of amphetamine can cause a decrease in RBC count and hemoglobin levels due to drug-induced stress on the body. In contrast, cannabis abuse has been shown to have minimal effects on blood indices. Some studies have reported slight decreases in RBC count and hemoglobin levels, but these changes are usually within the normal range and not clinically significant^4,5,20^. Our results are consistent with these studies showing the possible negative consequences of drug misuse on immunity. As shown in Table 2, there were significant changes in different leukocyte blood types, including neutrophils (NE; *p* value = 0.001), lymphocytes (LYM; *p* value = 0.001), monocytes (MON; *p* value = 0.002), eosinophils (EO; *p* value = 0.001), and basophils (BASO; *p* value = 0.001). Additional research revealed that the immunosuppressive effects of drug compounds could not be directly attributable to the drugs' effects on immune cells but rather may be explained by the production of endogenous immunomodulatory chemicals such as cytokines, free radicals, etc.^4–11,11–27^.

In addition, the microscopic view of blood film for patients who abuse the mixing drugs “Amphetamine and Cannabis”, shows segmented neutrophils with toxic change (Fig. 1B,C) have chromatin that is uneven and less condensed in the lobes, as well as a large number of large purple or dark blue cytoplasmic granules (primary granules) brought on by the retention of ribosomal RNA and cytoplasmic vacuolization. As opposed to their abnormal counterparts, normally matured segmented neutrophils in Fig. 1A (control) possess comparatively small nuclei, chromatin that is densely packed, and white cytoplasm with a majority of fine pink secondary granules. The exact cause of neutrophil toxicity is not fully understood, but several theories include direct toxic effects on bone marrow cells, activation of the immune system, and abnormal maturation of neutrophils. These effects can lead to nuclear granulation, which is not exclusive to cannabis and amphetamine abuse but can also occur in other conditions like infections and autoimmune diseases. Cytokines, primarily correlated with C-reactive protein, can cause toxic changes in neutrophils. Furthermore, it may be suggested that it is due to oxidative stress, which is characterized by the release of excess free radicals, reactive oxygen species, and cytokines and can lead to neutrophil cell destruction, causing cytotoxicity^28,29^.

Amphetamine abuse has been associated with liver damage, primarily due to the increased release of stress hormones and oxidative stress caused by the drug. Prolonged amphetamine abuse can lead to hepatotoxicity, characterized by elevated liver enzymes such as alanine aminotransferase (ALT) and aspartate aminotransferase (AST). Cannabis abuse, on the other hand, has shown conflicting results in terms of its effects on the liver profile. Some studies suggest a minor increase in liver enzymes, such as ALT and AST, in cannabis users. However, these changes are typically mild and not associated with severe liver damage^30^. Our results suggest no significant difference was observed in AST-GOT, ALT-GPT, and alkaline phosphatase levels, as depicted in Table 3, further validating the previously reported results^30^.

Amphetamine abuse has been linked to kidney damage, largely due to its vasoconstrictive effects and increased release of stress hormones. Long-term abuse of amphetamines can lead to renal impairment, characterized by elevated serum creatinine levels and a decreased glomerular filtration rate (GFR). In contrast, cannabis abuse has shown a limited impact on the kidney profile. Studies have been inconclusive, with some reporting a potential association between long-term cannabis use and mild kidney dysfunction, while others have found no significant effects^31^. Further validate our finding that neither creatinine nor blood urea nitrogen (BUN) levels were significantly affected among abusers (Table 3).

It is alarming to see that, in a fungus culture test using sputum samples from amphetamine and cannabis users, the majority of the samples (35 cultures) showed positive growth while just 15 cultures showed negative growth. Additionally, a few of the positive colonies are later stained with “Crystal Violet” and put under a microscope for inspection. A single or group of oval-shaped cells with budding features is seen in Fig. 2d; this is an example of a unicellular fungus that may suggest that due to the suppressed immunity, observed with blood results in opportunistic pathogens like yeast and mold inhabiting the upper respiratory tract of these chronic immunocompromised patients of drug abuse. However, further studies should be performed to identify the specific type. Our finding further validates the concept of "fungal priority pathogens", published by WHO recently (2022) which includes 19 groups of fungal pathogens that cause high-risk morbidity and mortality and aims to direct research to increase public health measures against invasive fungal diseases (IFDs)^21^.

The research aims to improve drug abuse research, diagnostics, treatment, and public health. It highlights that chronic amphetamine abuse has more severe effects on blood indices, liver profile, and kidney profile than cannabis abuse. However, individual variations in drug abuse patterns and susceptibility may influence these effects.

## Conclusion

This study explores the immune system and hematological disturbances linked to amphetamine and cannabis abuse, providing insights into health risks and targeted interventions. The findings complement previous research on drug users' hematological abnormalities, particularly neutrophil cytotoxicity in differential white blood counts. Routine hematological tests (CBC) help identify alterations in homeostatic conditions, improving patient knowledge and preventing major issues, may suggest specific screening protocols or monitoring strategies for individuals known to abuse these substances. The multifaceted approach needed to address substance abuse effectively in future research targeted multi-drug abuse prevention, early detection, and intervention. Addressing the limitation of the present study with a larger sample size with the addition of a control group in the study could further enhance the study's outcomes.

At present this cross-sectional design allows for a snapshot of the immune system and hematological status among abusers, laying the groundwork for future longitudinal research studies, that would allow for the assessment of long-term effects of substance abuse on the immune system and hematological parameters, providing insights into the progression of potential health issues and the effectiveness of the intervention. Besides the potential policy implications and efforts to combat drug abuse by the Saudi Government, there is a need to have more stress on drug education programs and support services for substance abusers.

## Data Availability

The datasets generated and/or analyzed during the current study are not publicly available for upholding the confidentiality of participants' data, but are available from the corresponding author on reasonable request.
